# Phenotypic and genotypic characterization of *Acinetobacter junii* and *Acinetobacter nosocomialis* isolated from humans, animals, and the environment in Lagos, Nigeria

**DOI:** 10.1016/j.onehlt.2026.101388

**Published:** 2026-03-12

**Authors:** Samuel O. Ajoseh, Abdul-Azeez A. Anjorin, Hanka Brangsch, Heinrich Neubauer, Gamal Wareth, Kabiru O. Akinyemi

**Affiliations:** a Department of Microbiology, Faculty of Science, Lagos State University, P.M.B 0001, Ojo, Lagos, Nigeria; b Institute of Bacterial Infections and Zoonoses, Friedrich-Loeffler-Institut (FLI), 07743 Jena, Germany; c Institute of Infectious Diseases and Infection Control, Jena University Hospital, 07747 Jena, Germany

**Keywords:** *Acinetobacter*, Antimicrobial resistance, Whole genome sequencing, Virulence factors, One health, Phylogenomic

## Abstract

**Background:**

The emerging threat of multidrug-resistant (MDR) *Acinetobacter* (*A.*) species, coupled with limited data on its prevalence, resistance mechanisms, and genomic profiles in Nigeria, necessitates immediate study.

**Aim:**

This investigation characterized the microbial traits and genetic variation of *Acinetobacter* species isolated from human, animal, and environmental sources, with emphasis on resistance dynamics, virulence, inter-source relatedness and diversity in Lagos, Nigeria.

**Methods:**

From April 2023 to March 2024, 2835 samples were collected (1410 human, 1020 animal, and 405 environmental), processed, and the isolates were identified by classical microbiological methods. Further identification was carried out by Matrix-Assisted Laser Desorption Ionization-Time of Flight (MALDI-TOF) mass spectrometry and confirmed by Whole Genome Sequencing (WGS). Antibiotic susceptibility testing (AST) was assessed by the microdilution method using the MICRONAUT system. WGS data were utilized for analyzing virulence factors, antimicrobial resistance (AMR) determinants, and phylogenetic lineages.

**Results:**

Nineteen isolates were confirmed as *Acinetobacter* by MALDI-TOF and WGS, of which 14 were *A. nosocomialis* and five were *A. junii*. The AST results revealed that both *Acinetobacter* species exhibited 100% resistance to cefotaxime and fosfomycin, while 100% of *A. nosocomialis* were resistant to chloramphenicol. Genomic analysis revealed that all *A. nosocomialis* isolates harbored 21 intrinsic AMR genes, including four distinct *bla*_ADC_ variants and 17 efflux-related genes, which were all absent in *A. junii*. Moreover, *A. nosocomialis* harbored 68 virulence genes spanning seven mechanisms, compared to six virulence genes spanning five mechanisms in *A. junii,* with the absence of exotoxin and biofilm formation mechanisms. Both species have six conserved virulence-associated genes [*ompA, ACICU_RS00500, bfmR, pilG, pilT,* and *vgrG/tssI*], and two distinct plasmids were exclusively detected in *A. nosocomialis*. Phylogenomic analysis demonstrated that both species formed distinct sub-clusters from different sources, suggesting shared evolutionary or transmission contexts. For example, five isolates (three *A. nosocomialis* from cattle nasal swab, human sputum, and abattoir effluent) and two *A. junii* from cattle rectal swab, and cattle nasal swab) form a distinct cluster differing by 15 SNP, indicating potential cross-species dynamics warranting deeper investigation.

**Conclusion:**

This study revealed the existence of *A. nosocomialis* and *A. junii* resistant to cefotaxime and fosfomycin in the One Health sector in Lagos. Both species were found to exhibit six conserved virulence genes linked to pathogenicity. Advanced molecular diagnostics is needed to monitor the emergence and spread of virulence and antibiotic-resistance in *Acinetobacter* species in Nigeria.

## 1 Introduction

*Acinetobacter* (*A*.) species are aerobic, non-fermentative Gram-negative coccobacilli that are ubiquitously present in soil and water, where they thrive as free-living saprophytes [1]. Additionally, certain species have been identified as commensal organisms that inhabit the skin, throat, and secretions of healthy individuals [2]. These opportunistic pathogens are known to colonize the hospital environment, including bed rails, floors, ventilator pads, supply carts, and infusion pumps in Intensive Care Units (ICUs) [3]. Among the over 118 identified *Acinetobacter* species (https://www.bacterio.net/genus/*Acinetobacter*/ accessed 14th October 2025), four are of particular clinical significance: *A. baumannii, A. pittii*, *A. nosocomialis,* and *A. junii* [4], [5]. These clinically significant species are part of a biochemically indistinguishable group known as the *Acinetobacter baumannii*-*calcoaceticus* (ABC) complex which also includes *A. seifertii, A. lactucae (*also known as *A. dijkshoorniae),* and *A. calcoaceticus* [6]. The biochemical similarities among these species initially led to confusion in their characterization and determination of clinical relevance [6]. However, the availability of advanced molecular tools has clarified the differences between these species [4], [6]. The accurate identification of *Acinetobacter* isolates at the species level presents significant challenges when employing standard phenotypic methods [7]. Matrix-Assisted Laser Desorption Ionization-Time of Flight (MALDI-TOF) mass spectrometry for microbial protein profiling [8], [9] and genotypic approaches, including partial *rpoB* sequencing [10] or ribosomal MLST analysis [11], have been utilized for *Acinetobacter* species classification. However, accurately distinguishing between the ABC complex species is still challenging. Among *Acinetobacter* species*, A. baumannii* is responsible for most infections attributed to the genus *Acinetobacter* due to its association with a broad spectrum of infections within ICUs [12]. However, *A. nosocomialis*, *A. pittii,* and *A. junii* are increasingly recognized as common causes of hospital-acquired infections (HAIs) [13], [14], [15]. These pathogens are notable for their ability to develop multiple resistance mechanisms against major antibiotic classes, posing a challenge in managing *Acinetobacter*-associated nosocomial infections [16]. They exhibit resistance to broad-spectrum β-lactams (such as 3^rd^ cephalosporins, carboxypenicillins, and, increasingly, carbapenems) [16]. Globally, both baumannii and non-baumannii *Acinetobacter* species have been increasingly detected in animal production and food-preparation environments, where their environmental persistence, biofilm formation, and carriage of antimicrobial-resistance determinants enable survival on farms, in processing facilities, and on foodstuffs, thereby posing One Health transmission risks from animals and food to humans [17], [18], [19].

On the other hand, *A. nosocomialis* [20] and *A. junii* [21] have been identified in environmental settings in Nigeria, however, detailed investigations into their epidemiology and molecular diversity in clinical and animal settings remain markedly limited. On this note, whole genome sequencing (WGS) offers high discriminatory power and accuracy in identifying strains of *Acinetobacter* species, facilitating the tracking of transmission pathways and the implementation of targeted interventions for these pathogens. In Nigeria, few studies have reported molecular epidemiological diversity of the genus *Acinetobacter* using WGS [22], [23]. There is a knowledge gap on the genomic characteristics of *Acinetobacter* species in Nigeria and other low- and middle-income countries, primarily due to limited research infrastructure and resources. In light of the transnational nature of antimicrobial resistance, it is imperative to investigate the epidemiological and genomic data to assess the burden of these pathogens and track antimicrobial resistance. This is crucial for developing effective infection control strategies and antibiotic stewardship interventions in Nigeria. This study aimed to determine the prevalence, antibiotic resistance, mechanism of resistance, clonal relatedness, and diversity of clinically relevant *Acinetobacter* species recovered from human, animal, and environmental sources in Lagos, Nigeria.

## 2 Materials and methods

### 2.1 Study area

**Fig. 1:**
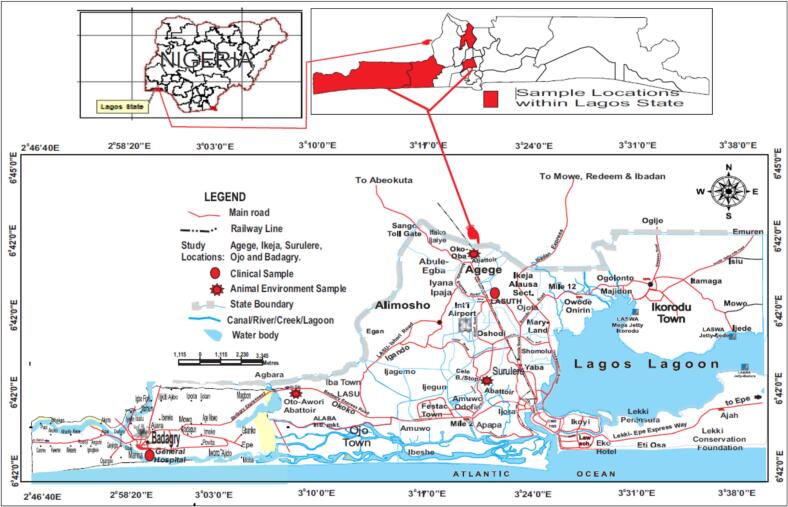
Spatial Mapping of Sample Collection Sites in Lagos, Nigeria.

The study was conducted in Lagos State, Nigeria. The study sites encompass key locations within the Lagos West Senatorial District, selected for their relevance to clinical and zoonotic disease potential (Fig. 1). These sites included the Lagos State University Teaching Hospital (LASUTH), Ikeja (Latitude: 6.5895°N, Longitude: 3.3422°E), and Badagry General Hospital (BGH), Badagry (Latitude: 6.4150°N, Longitude: 2.8813°E). Additionally, samples were obtained from two major abattoirs: Oto-Awori Abattoir, Badagry (Latitude: 6.4667°N, Longitude: 3.1833°E), and Oko-Oba Abattoir, Agege (Latitude: 6.6167°N, Longitude: 3.3333°E). LASUTH and BGH provided access to human samples from diverse demographics, while the abattoirs offered an opportunity to investigate zoonotic transmission dynamics in livestock and surrounding environments.

### 2.2 Ethical approval

Ethical approval for the use of clinical specimens was obtained from the Human Research and Ethics Committee of Lagos State University Teaching Hospital (LREC/06/10/1803) and from the Lagos State Health Service Commission (LSHSC/2222/VOL III/68). Patient recruitment commenced only after these clearances were secured, in full compliance with the principles of the Declaration of Helsinki (1964) and its later revisions. Written informed consent was obtained from each participant and, where applicable, from their legally authorized guardians. Ethical authorization for collecting animal specimens was granted by the Department of Veterinary Services, Ministry of Agriculture, Lagos State, Alausa (Health Research and Ethics Committee reference LREC/06/10/1803). All procedures were conducted in accordance with this approval and with prevailing animal welfare regulations. Biological samples (identified isolates) were sent to the Institute of Bacterial Infection and Zoonoses (IBIZ), Friedrich-Loeffler Institut (FLI), Jena, Germany in accordance to the Nagoya Protocol, with approval from the Federal Ministry of Environment, Abuja, Nigeria.

### 2.3 Study design

Between April 2023 and March 2024, a total of 2835 samples were collected, encompassing 1410 human samples, 1020 animal samples, and 405 environmental samples. The human samples were obtained from inpatients receiving treatment at two public referral hospitals, including Lagos State University Teaching Hospital (LASUTH) and Badagry General Hospital (BGH). Patients' medical histories and demographic information were documented. The patients were divided into two distinct cohorts. Group A consisted of 810 individuals having critical health conditions necessitating their admission to the medical emergency unit (MEU), surgical emergency unit (SEU), or intensive care unit (ICU) in LASUTH. The criteria for critical health conditions in MEU patients included severe symptoms such as chest pain, respiratory distress, altered mental status, severe infections, or other acute health issues. SEU patients required urgent surgical intervention for conditions such as traumatic injuries, acute abdominal pain, and appendicitis. ICU patients presented with life-threatening conditions, severe organ dysfunction, or complex medical needs, including respiratory failure, sepsis, cardiac emergencies, major surgical procedures, and trauma. In contrast, Group B comprised 600 participants who were not admitted into MEU, SEU, or ICU but were clinically diagnosed with chronic septicemia, liver diseases, kidney diseases, and respiratory-related illnesses in BGH. The sample size for clinical sample collection was determined using a formula typically employed for descriptive studies: N = Z^2^p(1–p)/d^2^, where N represents the required sample size, Z corresponds to the critical value at a 95% confidence interval (approximately 1.96 for large samples), d represents the desired level of precision (5% or 0.05), and p denotes disease prevalence (3.37%) as previously reported by Odewale et al. [24]. By substituting the provided values, N was determined to be approximately 108.8398885, which is roughly 108 samples per 100,000 individuals. To minimize biases and enhance accuracy, a total of 1410 human samples, including blood, sputum, oral swabs, urine, and wound swabs, were collected.

Veterinary doctors aseptically collected 1020 cattle samples (310 nasal and 310 rectal swabs) at the Oko-Oba abattoir in Agege, and 400 swabs (200 nasal and 200 rectal) at the Oto-Awori abattoir in Badagry, both in Lagos State. In addition, 405 environmental effluent samples were obtained aseptically, comprising 255 from Oko-Oba and 150 from Oto-Awori.

### 2.4 Collection and processing of samples

Human samples: Phlebotomists aseptically obtained blood samples from patients categorized into Groups A and B. Additionally, swabs were collected from the nose, throat, groin, perineum, and axillae, as well as sputum, catheter urine, and wound samples based on the patient's clinical complications.

Animal and environmental samples: Samples, including nasal swabs, and rectal swabs, as well as effluents and lagoon water, were also aseptically collected and transported under a cold chain to the research laboratory at the Department of Microbiology, Lagos State University, Ojo.

The blood samples were inoculated into blood culture bottles containing brain heart infusion (BHI) broth (Mast, UK) and gently mixed. The culture bottles were incubated aerobically at 37 °C for seven days. Turbid bottles were subsequently sub-cultured on McConkey agar (MA) (HIMEDIA, Mumbai, India) and further incubated for 18–24 h at 37 °C. Non-turbid blood culture bottles were also sub-cultured daily from the BHI broth for up to seven days, after which the blood-broth suspension was discarded. However, other clinical samples and non-clinical samples were inoculated on McConkey agar (MA) (HIMEDIA, Mumbai, India) and incubated for 18–24 h at 37 °C. Distinct colonies were sub-cultured on McConkey agar (MA) (HIMEDIA, Mumbai, India), further incubated for 18–24 h at 37 °C for pure culture isolation, and subjected to pre-confirmation using the API 20 N kit (Biomeriux, Marcy-l'Étoile, France).

### 2.5 Confirmatory identification of *Acinetobacter* species

A total of 41 suspected *Acinetobacter* species strains, isolated from diverse sources in Lagos, Nigeria, were sent to the Institute of Bacterial Infections and Zoonoses (IBIZ) in Friedrich-Loeffler Institut (FLI), Jena, Germany, for species confirmation and typing. The genetic materials were shared in agreement with the Nagoya Protocol, approved by the Federal Ministry of Environment, Abuja, Nigeria. Species-level identification was performed using matrix-assisted laser desorption/ionization mass spectrometry (MALDI-TOF MS, Bruker Daltonik MALDI Biotyper) with log scores >2.300, as previously reported [25]. Whole genome sequencing (WGS) and subsequent analysis were employed to confirm the identity of the strains using Kraken2 [26], [27].

### 2.6 Antimicrobial susceptibility testing

The minimum inhibitory concentration (MIC) of antibiotics for the strains was determined using the broth microdilution method in Mueller-Hinton II broth, with the automated MICRONAUT-S system (Micronaut, MERLIN Diagnostics GmbH, Bornheim-Hersel, Germany), following the manufacturer's guidelines. This method involves rehydrating antibiotic compounds by adding a standardized bacterial suspension, followed by incubation at 37 °C for 18–24 h. The MIC values were interpreted according to Clinical and Laboratory Standards Institute (CLSI) breakpoint guidelines for *Acinetobacter* species [28] as previously reported by Wareth et al. [29]. The susceptibility of all isolates was evaluated against a panel of 17 antibiotics: ciprofloxacin (CIP), levofloxacin (LEV), amikacin (AMK), colistin (COL), chloramphenicol (CMP), fosfomycin (FOS), tigecycline (TGC), trimethoprim/sulfamethoxazole (T/S), piperacillin (PIP), piperacillin/tazobactam (PIT), cefotaxime (CTX), ceftazidime (CAZ), ceftazidime/avibactam (CAA), ceftolozane/tazobactam (CTA), imipenem (IMP), meropenem (MER) and meropenem plus (MERM).

### 2.7 WGS analysis

DNA extraction was performed using the High-Pure template preparation kit (Roche Applied Sciences, Mannheim, Germany), according to the manufacturer's protocol. The 19 confirmed Nigerian *Acinetobacter* species strains were then subjected to library preparation and paired-end sequencing on an Illumina MiSeq sequencer and a Nanopore sequencer. The Nextera XT DNA Library Prep Kit (Illumina, Inc., San Diego, CA, USA) was employed for Illumina sequencing library construction, while the Native Barcoding Kit (SQK-NBD114.24) (Oxford Nanopore Technologies, Oxford, United Kingdom) was used for Nanopore sequencing. Nanopore libraries were run using R10.4.1 flow cells on a MinION MkB1 (Oxford Nanopore Technologies, Oxford, United Kingdom) for 72 h. Both Illumina and Nanopore assemblies of the sequenced 19 *Acinetobacter* species were considered for bioinformatics analysis.

Additionally, raw sequencing data were downloaded from the National Center for Biotechnology Information's Sequence Read Archive (SRA) (accessed on 21 February 2025) according to the following criteria: Search for species [*A. nosocomialis, A. junii*] with the geo_loc_name_country_continent [Nigeria, Africa, Europe, Asia]. The results were filtered, and the search items following the Library Layout [paired], Library Source [genomic], and Platform [Illumina] were eligible for inclusion. The sequence data of 63 *A. nosocomialis* strains and 43 *A. junii* strains were used, along with the isolates recovered in this study, for phylogenetic analysis.

### 2.8 Bioinformatics analysis

Long-read basecalling was performed using Dorado v0.9.1 (https://github.com/nanoporetech/dorado). Taxonomic classification of the sequences was conducted utilizing Kraken2 v2.1.3 [30]. For hybrid genome assembly, BONT v1 [31] was employed, using Unicycler v0.5.0 [31] and Polypolish v0.6.0 [32]. Genome assembly statistics were assessed using QUAST v5.2.0 [33]. BUSCO v5.8.3 [34] was used for completeness assessment. Furthermore, the sequenced species identity was confirmed by checking the average nucleotide identity (ANI) to reference strains of *A. junii* (*n* = 10) and *A. nosocomialis* (n = 10) using fastANI v1.34 [35]. Finally, genome annotation was conducted using Bakta v1.10.3 [36] with database v5.1.

### 2.9 Antimicrobial resistance (AMR) genes, virulence genes, and plasmid typing analysis

Gene screening was performed with ABRicate v1.0.1 (https://github.com/tseemann/abricate), utilizing multiple databases including Vfdb setA (08.03.2025) [37], NCBI [38], CARD [39], and ResFinder [40] to comprehensively identify resistance and virulence determinants [41]. For AMR screening, AMRfinder Plus v3.11.26 [38] with the —O option was employed. Plasmid typing specific to *Acinetobacter* species was conducted using *Acinetobacter* plasmid typing v3.0 [42], leveraging the latest available database version from February 2025 to classify and characterize plasmid-associated sequences.

### 2.10 Comparative genomic and phylogenetic analysis

For comparative genomic analyses, nucleotide variant calling was performed using Snippy v4.6.0 (https://github.com/tseemann/snippy) [43], incorporating five *A. junii* isolates, a reference genome (GCF_018336855.1), and 42 publicly available *A. junii* sequence genomes randomly selected from global datasets in public repositories. Similarly, analysis was conducted on 14 *A. nosocomialis* isolates, a reference genome (GCF_041021905.1), and 62 publicly accessible *A. nosocomialis* sequence datasets. Phylogenetic relationships among the strains were inferred using maximum likelihood analysis implemented in RAxML v8.2.12 [44] with the parameters recommended for SNP alignments by the manual (−m ASC_GTRCAT –asc-corr = lewis -V —N autoMRE), i.e. without correction for rate heterogeneity, with standard ascertainment bias correction by Paul Lewis and automatic choice of number of bootstrapping replicates. The resulting tree was visualized using Microreact [45].

## 3 Results

### 3.1 Identification of *Acinetobacter* species

From a total of 2835 diverse-source samples collected in Lagos, Nigeria, 41 (11 human, 16 animal, and 14 environmental) isolates were preliminarily identified as *Acinetobacter* species using classical bacteriology. Of them, 19 isolates (9 human, 8 animal and 2 from environmental sources) were identified as *Acinetobacter* species using MALDI-TOF mass spectrometry (score range: 1.7–2.51) and whole genome sequencing (WGS) (Table 1). 22 isolates were misidentified by classical bacteriology as *Acinetobacter*. However, MALDI-TOF suscessfully identified them as *Stenotrophomonas maltophilia* (*n* = 4), *Achromobacter dentrificans* (*n* = 7), *Ochrobactrum tritici* (*n* = 1), *Ochrobactrum intermedium* (*n* = 3), *Achromobacter mucicolens* (*n* = 2), and *Achromobacter insolitus* (*n* = 5) (Table 2). Those bacteria were derived from human clinical samples (blood, sputum, and oral swabs), cattle specimens (nasal swabs and rectal swab), and environmental samples, including abattoir effluents.

**Table 1:** *Acinetobacter* species detected in different sources in Lagos, Nigeria, using API, MALDI-TOF, and WGS methods.

Sample Code_GER	Location	Date of Collection	Source	Sample Type	Sex	Age	Prognosis	API ID	MALDI-TOF Score value	WGS % 1st Match		*Acinetobacter* species
										Genus	Species	
24AR27661	OOA	2/10/2023	Cattle	Nasal swab	M	3	Ready-to-Slaughter	ND	2.04	97.98	70.95	*A. nosocomialis*
24AR27662	OOA	2/10/2023	Cattle	Nasal swab	M	2	Ready-to-Slaughter	ND	2.17	96.64	65.57	*A. nosocomialis*
24AR27663	OAA	19/9/2023	Cattle	Nasal swab	M	3	Ready-to-Slaughter	ND	2.25	98.24	71.57	*A. nosocomialis*
24AR27665	OAA	19/9/2023	Cattle	Rectal swab	M	3	Ready-to-Slaughter	ND	2.42	95.44	78.77	*A. junii*
24AR27668	OAA	19/9/2023	Cattle	Nasal swab	M	3	Ready-to-Slaughter	ND	2.51	96.28	80.09	*A. junii*
24AR27669	OAA	19/9/2023	Cattle	Nasal swab	M	3	Ready-to-Slaughter	ND	2.49	96.12	80.44	*A. junii*
24AR27700	OAA	19/9/2023	Cattle	Rectal swab	M	2	Ready-to-Slaughter	ND	2.42	85.61	54.32	*A. nosocomialis*
24AR27776	OAA	19/9/2023	Cattle	Nasal swab	M	3	Ready-to-Slaughter	ND	1.7	98.17	71.03	*A. nosocomialis*
24AR27701	OOAE	7/3/2023	Environment	Effluent	ND	ND	ND	ND	2.47	95.64	79.35	*A. junii*
24AR27704	OAAE	23/1/2023	Environment	Effluent	ND	ND	ND	ND	2.12	97.25	68.61	*A. nosocomialis*
24AR27683	BGH	18/1/2023	Human	Blood	F	46	Sepsis	ND	2.33	87.37	54.06	*A. nosocomialis*
24AR27706	BGH	1/23/2023	Human	Blood	F	55	Sepsis	ND	2.08	96.46	68.64	*A. nosocomialis*
24AR27770	BGH	23/1/2023	Human	Blood	F	27	Sepsis, Liver disease	ND	2	97.86	70.43	*A. nosocomialis*
24AR27771	BGH	23/1/2023	Human	Blood	F	33	Sepsis, Liver disease	ND	2.22	98.06	71.62	*A. nosocomialis*
24AR27772	BGH	23/1/2023	Human	Sputum	M	31	Sepsis, Respiratory disease, Liver disease	ND	2.19	98.05	70.73	*A. nosocomialis*
24AR27773	BGH	23/1/2023	Human	Oral swab	M	2	Sepsis, Liver disease	ND	2.06	98.13	72.41	*A. nosocomialis*
24AR27774	BGH	23/1/2023	Human	Sputum	M	7	Respiratory diseases	ND	2.4	92.28	58.25	*A. nosocomialis*
24AR27791⁎	LASUTH	31/1/2023	Human	Urine	F	25	Cather-associated kidney disease	ND	2.41	95.54	78.83	*A. junii*
24AR27892	BGH	18/1/2023	Human	Blood	F	36	Sepsis	ND	2.26	97.88	70.5	*A. nosocomialis*

**Keys:** OOA: Oto-Awori Abattoir, OAA: Oko-Oba Abattoir, OOAE: Oko-Oba Abattoir effluents, OAAE: Oto-Awori Abattoir effluents, BGH: Badagry General Hospital, LASUTH: Lagos State University Teaching Hospital, ND: Not determined, M: Male, F: Female. Age in years.

⁎ Isolate obtained from the ICU at LASUTH – Group A.

**Table 2:** Other bacterial species isolated from animal, human and environmental sources from different sources in Lagos, Nigeria, but confirmed as non-*Acinetobacter* using MALDI-TOF.

Sample source	Source Type	Location	FLI code	NIG_code	MALDI-TOS (score 1.5–2.8) identification name
Animal	Nasal swab	Oto-Awori	24AR27664	OAN4A	*Stenotrophomonas maltophilia*
	Rectal swab	Cele	24AR27787	CCRS12A	*Stenotrophomonas maltophilia*
	Rectal swab	Oko-oba	24AR27671	KNS06A	*Achromobacter dentrificans*
	Nasal swab	Oto-Awori	24AR27672	OAN12A	*Achromobacter dentrificans*
	Rectal swab	Oto-Awori	24AR27673	OAM10B	*Ochrobactrum tritici*
	Nasal swab	Oto-Awori	24AR27768	OAN14B	*Achromobacter denitrificans*
	Nasal swab	Oto-Awori	24AR27676	OAN13	*Ochromobacter intermedium*
	Rectal swab	Cele	24AR27678	CCRS12A	*Achromobacter denitrificans*
Human	Blood	Badagry	24AR27681	BGB019	*Achromobacter mucicolens*
	Blood	Badagry	24AR27684	BGB015A	*Achromobacter insolitus*
	Urine	Badagry	24AR27685	BGU059	*Achromobacter denitrificans*
	Oral swab	Lasuth	24AR27687	LAO005	*Achromobacter insolitus*
	Wound swab	Lasuth	24AR27689	LAW034	*Ochromobacter intermedium*
	Urine	Lasuth	24AR27692	LAU031	*Ochromobacter intermedium*
	Urine	Lasuth	24AR27693	LAU025A	*Achromobacter insolitus*
	Blood	Badagry	24AR27694	BGB118	*Achromobacter denitrificans*
	Blood	Lasuth	24AR27695	LAB059	*Achromobacter mucicolens*
	Sputum	Badagry	24AR27696	BGS006A	*Achromobacter insolitus*
	Urine	Lasuth	24AR27697	LAU003	*Achromobacter insolitus*
Environment	Masa-Masa Lagoon	Masa-Masa	24AR27699	M22	*Achromobacter denitrificans*
	Abattoir effluent	Cele	24AR27702	CAE08A	*Stenotrophomonas maltophilia*
	Abattoir effluent	Cele	24AR27706	CAE01	*Stenotrophomonas maltophilia*

Among 19 *Acinetobacter* species isolates confirmed in this study, *A. nosocomialis* was most frequently isolated, accounting for 14 (62.5%) of the recovered *Acinetobacter* isolates. Specifically, 8 isolates (57%) were obtained from human samples, while five (36%) originated from animal sources, and one (7%) was recovered from environmental samples. Additionally, *A. junii* represented 5 (26%) of the confirmed isolates, three (60%) were obtained from ready-to-slaughter cattle, one (20%) was of human origin and one (20%) obtained from environmental samples (Table 1).

### 3.2 Antimicrobial susceptibility and resistance of *Acinetobacter* species from diverse sources

The *Acinetobacter* spp. isolates showed complete resistance (100%) to cefotaxime and fosfomycin. It is noteworthy that 100% (14/14) of *A. nosocomialis* were resistant to chloramphenicol, while all *A. junii* (5/5) were susceptible to this antibiotic. All of the *Acinetobacter* spp. isolates were intermediately resistant to ciprofloxacin, while only one isolate (*A. junni* 24AR27669), recovered from ready-to-slaughter cattle at Oto-Awori Abattoir, was resistant to colistin. Interestingly, all isolates were susceptible to 12 of the 17 tested antibiotics, including levofloxacin, amikacin, tigecycline, trimethoprim/sulfamethoxazole, piperacillin, piperacillin/tazobactam, ceftazidime, ceftazidime/avibactam, ceftolozane/tazobactam, imipenem, meropenem, and meropenem plus (Table 3).

**Table 3:** Antimicrobial susceptibility and resistance of *Acinetobacter* species isolated from different sources in Lagos, Nigeria.

Sample Code_GER	Location	Source	Sample Type	Sex	Prognosis	Age	*Acinetobacter* species	Antibiotics Tested																
								CIP	LEV	AMK	COL	CMP	FOS	TGC	T/S	PIP	PIT	CTX	CAZ	CAA	CTA	IMP	MER	MERM
24AR27661	OOA	Cattle	NS	M	RTS	3	*A. nosocomialis*	I	S	S	S	R	R	S	S	S	S	R	S	S	S	S	S	S
24AR27662	OOA		NS	M	RTS	2	*A. nosocomialis*	I	S	S	S	R	R	S	S	S	S	R	S	S	S	S	S	S
24AR27663	OAA		NS	M	RTS	3	*A. nosocomialis*	I	S	S	S	R	R	S	S	S	S	R	S	S	S	S	S	S
24AR27665	OAA		Rectal swab	M	RTS	3.0	*A. junii*	I	S	S	S	S	R	S	S	S	S	R	S	S	S	S	S	S
24AR27668	OAA		NS	M	RTS	3	*A. junii*	I	S	S	S	S	R	S	S	S	S	R	S	S	S	S	S	S
24AR27669	OAA		NS	M	RTS	3	*A. junii*	I	S	S	R	S	R	S	S	S	S	R	S	S	S	S	S	S
24AR27700	OAA		Rectal swab	M	RTS	2.0	*A. nosocomialis*	I	S	S	S	R	R	S	S	S	S	R	S	S	S	S	S	S
24AR27776	OAA		NS	M	RTS	3	*A. nosocomialis*	I	S	S	S	R	R	S	S	S	S	R	S	S	S	S	S	S
24AR27701	CAE	Env.	Effluent	ND	ND	ND	*A. junii*	I	S	S	S	S	R	S	S	S	S	R	S	S	S	S	S	S
24AR27704	CAE		Effluent	ND	ND	ND	*A. nosocomialis*	I	S	S	S	R	R	S	S	S	S	R	S	S	S	S	S	S
24AR27683	BGH	Human	Blood	F	Sepsis	46	*A. nosocomialis*	I	S	S	S	R	R	S	S	S	S	R	S	S	S	S	S	S
24AR27706	BGH		Blood	F	Sepsis	55	*A. nosocomialis*	I	S	S	S	R	R	S	S	S	S	R	S	S	S	S	S	S
24AR27770	BGH		Blood	F	sepsis, LDs	27	*A. nosocomialis*	I	S	S	S	R	R	S	S	S	S	R	S	S	S	S	S	S
24AR27771	BGH		Blood	F	sepsis, LDs	33	*A. nosocomialis*	I	S	S	S	R	R	S	S	S	S	R	S	S	S	S	S	S
24AR27772	BGH		Sputum	M	sepsis, Kds, LDs	31	*A. nosocomialis*	I	S	S	S	R	R	S	S	S	S	R	S	S	S	S	S	S
24AR27773	BGH		OS	M	sepsis, LDs	2	*A. nosocomialis*	I	S	S	S	R	R	S	S	S	S	R	S	S	S	S	S	S
24AR27774	BGH		Sputum	M	RTD	7	*A. nosocomialis*	I	S	S	S	R	R	S	S	S	S	R	S	S	S	S	S	S
24AR27791⁎	LASUTH		Urine	F	CVP	25	*A. junii*	I	S	S	S	S	R	S	S	S	S	R	S	S	S	S	S	S
24AR27892	BGH		Blood	F	Sepsis	36	*A. nosocomialis*	I	S	S	S	R	R	S	S	S	S	R	S	S	S	S	S	S

**Key:** NS=Nasal sample, OS=Oral swab, M = male, F=Female, RTS = Ready-to-Slaughter, OOA = Oko-Oba Abattoir, OAA = Oto-Awori Abattoir, CAE = Cele Abattoir effluents, BGH=Badagry General Hospital, LASUTH = Lagos State University, CIP: ciprofloxacin, LEV: levofloxacin, AMK: amikacin, COL: colistin, CMP: chloramphenicol, FOS: fosfomycin, TGC: tigecycline, T/S: trimethoprim/sulfamethoxazole, PIP: piperacillin, PIT: piperacillin/tazobactam, CTX: cefotaxime, CAZ: ceftazidime, CAA: ceftazidime/avibactam, CTA: ceftolozane/tazobactam, CEP: cefepime, ERT: ertapenem, IMP: imipenem, and MER: meropenem.

⁎ Isolate obtained from ICU at LASUTH – Group A.

### 3.3 Assembly properties of *Acinetobacter* species

**Fig. 2:**
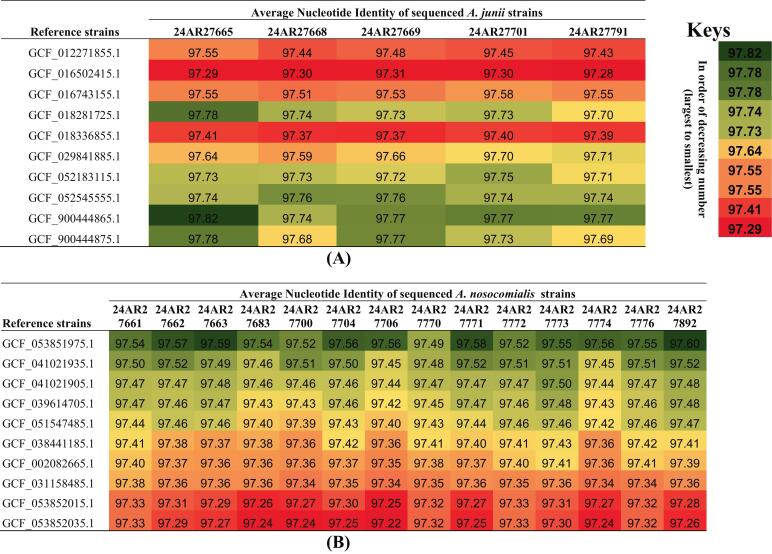
The heat map of average nucleotide identity of sequenced *Acinetobacter* species. (A) represents the five sequenced *A. junii* with ten reference strains; (B) represents the 14 sequenced *A. nosocomialis* with ten reference strains. Thick green colour = the highest ANI values; Yellow = mid ANI values; pale red = lower ANI values; thick red = lowest ANI value as indicated in the key. (For interpretation of the references to colour in this figure legend, the reader is referred to the web version of this article.)

Notably, of the two assemblies (Nanopore and Illumina), all five *A. junii* genomes were assembled as single contiguous sequences, with total lengths approximating 3.4 Mbp. In contrast, the *A. nosocomialis* genomes exhibited greater complexity, consisting of three to five contigs, with total lengths ranging from approximately 3.86 to 4.00 Mbp. The GC content was consistent (approximately 38.75%) across all 19 isolates. BUSCO analysis yielded high completeness scores, predominantly reaching 99.7%, with minimal fragmentation or missing genes. Furthermore, all pairwise average nucleotide identity (ANI) values of the five sequenced *A. junii* with the ten reference genomes fall between ∼97.28% and 97.82% (Fig. 2A), well above commonly used species thresholds (∼95–96%) [35]. This strongly supports that the five sequenced isolates (24AR27665, 24AR27668, 24AR27669, 24AR27701, 24AR27791) are *A*. *junii* and are correctly assigned at the species level. Within each reference genome, the ANI values across the five isolates vary only modestly (typical per-reference ranges ≈ 0.03–0.13%), and across references, each isolate's ANI spans ≈ 0.45–0.53%. These narrow ranges indicate the sequenced strains are genetically similar to one another and to the available reference genomes. However, some reference assemblies show consistently higher ANI values (e.g., GCF_900444865.1 reaches the dataset maximum of 97.8223% with strain 24AR27665), while others are slightly lower (e.g., GCF_016502415.1 values cluster near 97.28–97.31%). This pattern suggests small but measurable differences in relatedness among reference genomes and the sequenced isolates. Because ANI values are uniformly high and tightly clustered, the five isolates likely represent a coherent *A. junii* clade with limited genomic divergence from available references. Also, ANI values across all comparisons between the 14 *A. nosocomialis* and the 10 reference genomes range from approximately 97.22% (lowest, e.g., 24AR27706 vs GCF_053852035.1) to 97.60% (highest, e.g., 24AR27892 vs GCF_053851975.1) (Fig. 2B). This consistently high range (>97%) affirms that all 14 sequenced isolates are closely related to the reference strains, supporting their classification within *A. nosocomialis*. Specifically, the reference strain GCF_053851975.1 exhibits the highest ANI values with the sequenced isolates (mean ∼ 97.55%), suggesting it is the most genetically similar archetype. Isolates 24AR27663 (97.59%) and 24AR27892 (97.60%) show strong affinity (Fig. 2B). However, ANI values decrease progressively across the reference strains, with the lowest affinities observed for GCF_053852015.1 and GCF_053852035.1 (means ∼97.29% and ∼ 97.27%, respectively). This gradient may reflect intraspecific variation or subtle phylogenetic subclades within *A. nosocomialis*. Isolate-specific trends are evident; for example, 24AR27706 consistently shows lower ANI (e.g., ∼97.22–97.56%) than others, such as 24AR27892 (∼97.26–97.60%) (Fig. 2B).

### 3.4 *In-silico* detection of AMR genes in *Acinetobacter* spp. isolates

The *in-silico* analysis of AMR determinants identified 21 AMR-associated genes conferring resistance to 12 antibiotic classes. Of these, four AMR genes encoded resistance to a single antibiotic class (cephalosporins). These include *bla*_ADC_, *bla*_ADC-148_, *bla*_ADC-25_, and *bla*_ADC-68_, which encode for resistance against beta-lactam antibiotics (penicillin and cephalosporin) (Table 3). However, the remaining 17 genes were associated with multi-efflux systems, conferring resistance across multiple antibiotic categories (Table 4). Meanwhile, when using the National Center for Biotechnology Information (NCBI) database and ResFinder, a single gene was identified for each. None of the 21 AMR-associated genes were detected in *A. junii*. Conversely, 17 efflux-related genes and four AMR genes were present in 100% of *A. nosocomialis* isolates. These seventeen efflux-encoding genes are categorized into three major pump families: the small multidrug resistance (SMR) system, the *Ade* complex, and the major facilitator superfamily (MFS). Together, these pumps mediate resistance to a wide array of antibiotics, including aminoglycosides, fluoroquinolones, tetracyclines, macrolides, chloramphenicol, tigecycline, trimethoprim and carbapenems. Interestingly, all the identified genes were located on the chromosomal contig, indicating their intrinsic genomic origin. Specifically, all 14 *A. nosocomialis* isolates demonstrated an identical antimicrobial resistance phenotype (CMP–FOS–CTX) and harbored the same set of resistance determinants (*bla*_ADC_, *bla*_ADC-148_, *bla*_ADC-68_, and *bla*_ADC-25_). Of these isolates, eight originated from human clinical specimens, five from cattle, and one from an environmental source. In contrast, despite no AMR genes being detected in the five *A. junii* isolates, four of the five *A. junii* isolates shared a common resistance phenotype (FOS–CTX). A single *A. junii* isolate (24AR27669) exhibited a broader resistance profile (COL–FOS–CTX), including resistance to colistin.

**Table 4:** Antibiotic resistance profiles and resistance genes in *Acinetobacter* species from different sources.

Sample Code	Source	Sample Type	*Acinetobacter* species	Antibiotic Resistant Profile	AMR Genes
24AR27661	Cattle	Nasal Swab	*A. nosocomialis*	CMP-FOS-CTX	*bla*ADC-148, *bla*ADC, *bla*ADC-25, *bla*ADC-68
24AR27662		Nasal Swab	*A. nosocomialis*	CMP-FOS-CTX	*bla*ADC-148, *bla*ADC, *bla*ADC-25, *bla*ADC-68
24AR27663		Nasal Swab	*A. nosocomialis*	CMP-FOS-CTX	*bla*ADC-148, *bla*ADC, *bla*ADC-25, *bla*ADC-68
24AR27665		Rectal swab	*A. junii*	FOS-CTX	NIL
24AR27668		Nasal Swab	*A. junii*	FOS-CTX	NIL
24AR27669		Nasal Swab	*A. junii*	COL-FOS-CTX	NIL
24AR27700		Rectal swab	*A. nosocomialis*	CMP-FOS-CTX	*bla*ADC-148, *bla*ADC, *bla*ADC-25, *bla*ADC-68
24AR27776		Nasal Swab	*A. nosocomialis*	CMP-FOS-CTX	*bla*ADC-148, *bla*ADC, *bla*ADC-25, *bla*ADC-68
24AR27701	Env.	Effluent	*A. junii*	FOS-CTX	NIL
24AR27704		Effluent	*A. nosocomialis*	CMP-FOS-CTX	*bla*ADC-148, *bla*ADC, *bla*ADC-25, *bla*ADC-68
24AR27683	Human	Blood	*A. nosocomialis*	CMP-FOS-CTX	*bla*ADC-148, *bla*ADC, *bla*ADC-25, *bla*ADC-68
24AR27706		Blood	*A. nosocomialis*	CMP-FOS-CTX	*bla*ADC-148, *bla*ADC, *bla*ADC-25, *bla*ADC-68
24AR27770		Blood	*A. nosocomialis*	CMP-FOS-CTX	*bla*ADC-148, *bla*ADC, *bla*ADC-25, *bla*ADC-68
24AR27771		Blood	*A. nosocomialis*	CMP-FOS-CTX	*bla*ADC-148, *bla*ADC, *bla*ADC-25, *bla*ADC-68
24AR27772		Sputum	*A. nosocomialis*	CMP-FOS-CTX	*bla*ADC-148, *bla*ADC, *bla*ADC-25, *bla*ADC-68
24AR27773		Oral Swab	*A. nosocomialis*	CMP-FOS-CTX	*bla*ADC-148, *bla*ADC, *bla*ADC-25, *bla*ADC-68
24AR27774		Sputum	*A. nosocomialis*	CMP-FOS-CTX	*bla*ADC-148, *bla*ADC, *bla*ADC-25, *bla*ADC-68
24AR27791		Urine	*A. junii*	FOS-CTX	NIL
24AR27892		Blood	*A. nosocomialis*	CMP-FOS-CTX	*bla*ADC-148, *bla*ADC, *bla*ADC-25, *bla*ADC-68

### 3.5 *In-silico* detection of intrinsic virulence genes and plasmid type profiling

The analysis of virulence-associated genes identified a total of 68 genes encoding seven distinct virulence mechanism groups (exotoxin, immune modulation, nutrient/metabolic factor, regulatory, adherence, effector delivery system, and biofilm production) in *A. nosocomialis*. In contrast, *A. junii* exhibited only six virulence-associated genes, representing five of the seven mechanism groups, excluding exotoxin-encoding and biofilm genes. Within *A. nosocomialis*, the predominant virulence mechanism was adherence, accounting for 17 genes, followed by effector delivery systems (15 genes), immune modulation (10 genes), nutrition/metabolic factors (6 genes), and exotoxin production genes (3), with regulation being the least represented category (2 genes). Conversely, *A. junii* possessed a single gene for each of the five detected virulence mechanism groups:Adherence, effector delivery systems, immune modulation, nutrition/metabolic factors, and regulation, but lacked any gene associated with exotoxin production and biofilm. Interestingly, certain genes were universally present across all strains of *A. nosocomialis* and *A. junii* analyzed. Specifically, 100% of the *Acinetobacter* species strains harbored the *ompA* gene, which plays a pivotal role in immune modulation, contributing to biofilm formation, environmental persistence, and antibiotic resistance. Additionally, the *ACICU_RS00500* gene, associated with nutrition and metabolism, was consistently found across all genome sequences, as well as the *bfmR* gene, which encodes a regulatory mechanism, the *pil*G and *pil*T genes involved in adherence, and the *vgr*G/*tss*I gene responsible for effector delivery systems (Table 5).

**Table 5:** The mechanism and frequency of virulence genes detected in sequenced *Acinetobacter* species.

S/No.	Virulence Mechanism(n)	Virulence Genes (F)
1	Exotoxins (3)	*plc1*(14)*, plc2* (14)*, plcD* (14)
2	Immune modulation (10)	*lpxM* (14)*, lpxL*(14)*, lpsB* (14)*, lpxA* (14)*, lpxB (14), lpxC* (14)*, lpxD* (14)*, pbpG* (14)*, ompA* (19)*, tviB* (14)
3	Nutritional/Metabolic (6)	*galU* (14)*, pgi* (14)*, gale* (14)*, ACICU_RS00485* (14)*, ACICU_RS04565* (14)*, ACICU_RS00500* (19)
4	Regulation (2)	*bfmR* (19)*, bfmS* (14)
5	Adherence (17)	*PilM* (14)*, pilN* (14)*, pilO* (14)*, pilP* (14)*, pilT* (19)*, pilU* (14)*, pilF* (14)*, fimV* (14)*, pilB* (14)*, pilC* (14)*, tsaP* (14)*, fimT* (14)*, pilG* (19*), pilH* (14)*, pilI* (14)*, pilS* (14)*, pilR* (14)*,*
6	Biofilm (15)	*csuA/B* (14)*, csuA* (14)*, csuB* (14)*, csuC* (14)*, csuD* (14)*, csuE* (14)*, pgaA* (14)*, pgaB* (14)*, pgaC* (14)*, pgaD* (14)*, adeF* (14)*, adeG* (14)*, adeH*(14)*, abaI* (14)*, abaR* (14)
7	Effector delivery system (15)	*gspO/pilD* (14)*, gspN* (14)*, gspC* (14)*, gspD* (14)*, gspE1* (14)*, gspE2* (14)*, gspF* (14)*, gspH* (14)*, gspI* (14)*, gspK* (14)*, gspL* (14)*, gspM* (14)*, cpaA* (14*), vgrG/tssI* (19), *tse4* (14)

N: number; F: frequency.

Also, two plasmid types, R3-T21 and R3-T60, were identified in the 14 sequenced *A. nosocomialis.* R3-T21 was only missing in one isolate (24AR27662) recovered from ready-to-slaughter cattle in the Oko-Oba abattoir farm*.* No plasmid was detected in *A. junii* (Table 6). Interestingly, plasmid type R3-T21 was missing in Nanopore data-based assemblies of six *A. nosocomialis* strains but was detected in Illumina assemblies. It is worth noting that none of the AMR and virulence genes were found on predicted plasmid contigs. Specifically, all 14 *A. nosocomialis* strains uniformly harbor *bla*_ADC_ variants, encode 68 virulence genes across seven functional categories, and carry plasmids R3-T60 (100%) and R3-T21 (93%), exhibiting a consistent CMP–FOS–CTX resistance profile that heightens their potential for multisource outbreaks. In contrast, *A. junii* isolates from different sources in Lagos, Nigeria, coalesce into a single subclade despite varied origins, possess a reduced genomic repertoire devoid of detectable AMR genes and limited to six virulence determinants in five groups, yet display phenotypic FOS–CTX resistance, with strain 24AR27669 uniquely also resistant to COL reflecting a divergent adaptation strategy.

**Table 6:** Plasmid profiles of *Acinetobacter* species.⁎

Isolate			Plasmid Types	
	*Acinetobacter* species	No. detected	R3-T21	R3-T60
			100% sequence identity (or match)	
24AR27661	*A. nosocomialis*	2	100	100
24AR27662	*A. nosocomialis*	1	–	100
24AR27663	*A. nosocomialis*	2	100	100
24AR27665	*A. junii*	0	–	–
24AR27668	*A. junii*	0	–	–
24AR27669	*A. junii*	0	–	–
24AR27683	*A. nosocomialis*	2	100	100
24AR27700	*A. nosocomialis*	2	100	100
24AR27701	*A. junii*	0	–	–
24AR27704	*A. nosocomialis*	2	100	100
24AR27706	*A. nosocomialis*	2	100	100
24AR27770	*A. nosocomialis*	2	100	100
24AR27771	*A. nosocomialis*	2	100	100
24AR27772	*A. nosocomialis*	2	100	100
24AR27773	*A. nosocomialis*	2	100	100
24AR27774	*A. nosocomialis*	2	100	100
24AR27776	*A. nosocomialis*	2	100	100
24AR27791	*A. junii*	0	–	–
24AR27892	*A. nosocomialis*	2	100	100

“–” indicates no match.

⁎ Note: The shaded parts were missing in Nanopore-based assemblies.

### 3.6 Single-nucleotide polymorphism typing

**Fig. 3:**
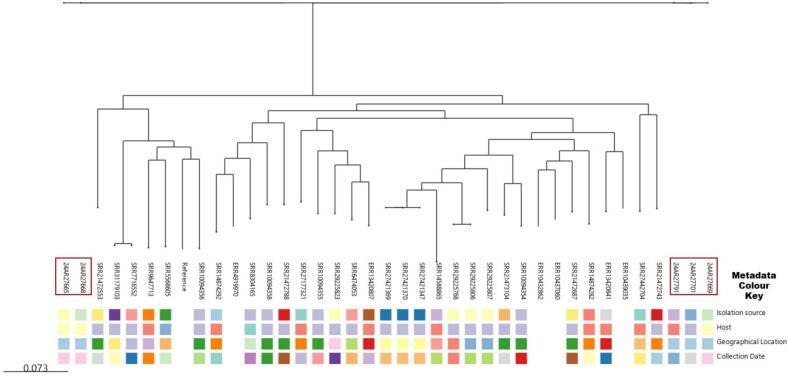

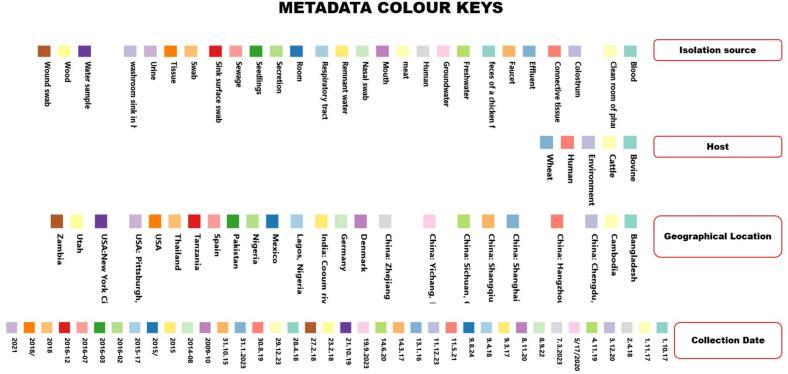
The phylogenetic tree of 5 sequenced *A. junii* and 43 deposited sequenced *A. junii* extracted from NCBI. The sequenced *A. junii* isolates are highlighted in red, others are public repository strains. The boxes below the strains are different the metadata of the both the sequenced strains and international strains including, isolation source, host, geographical locations and sample collection date. (For interpretation of the references to colour in this figure legend, the reader is referred to the web version of this article.)

**Fig. 4:**
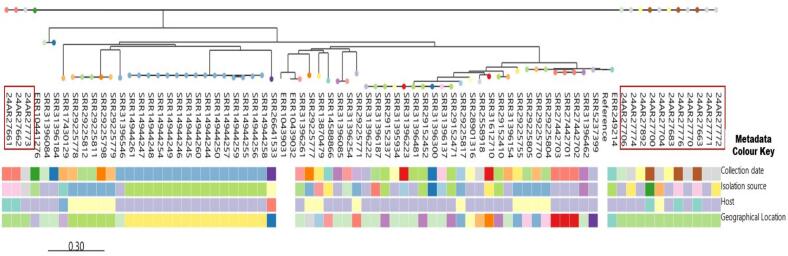

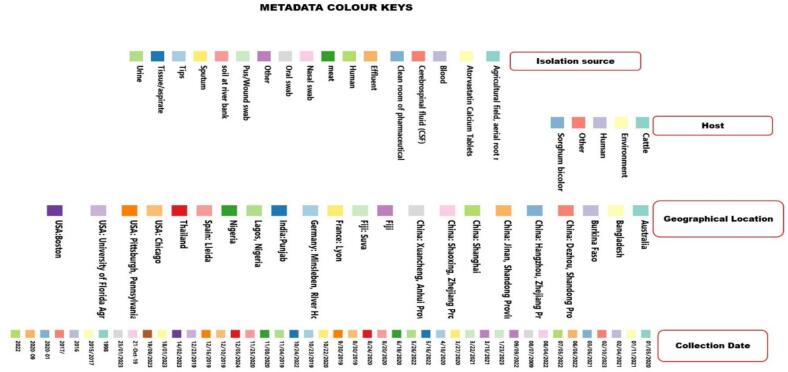
Phylogenetic tree of 14 sequenced *A. nosocomialis* and 63 deposited sequenced *A. nosocomialis* extracted from NCBI. The sequenced *A. nosocomialis* isolates are highlighted in red, others are public repository strains. The boxes below the strains are different the metadata of the both the sequenced strains and international strains including, isolation source, host, geographical locations and sample collection date. (For interpretation of the references to colour in this figure legend, the reader is referred to the web version of this article.)

Two independent phylogenetic trees were constructed for each species (*A. junii* and *A. nosocomialis*) to illustrate the similarity of the isolates based on single nucleotide polymorphisms (SNPs). The first tree, shown in Fig. 3, comprises five *A. junii* isolates (one from a human, three from animals, and one from abattoir effluents) and 43 global *A. junii* strains. The second tree (Fig. 4) includes 14 *A. nosocomialis* isolates recovered from diverse sources (five animal, one environmental, and eight human) and 63 international sequences obtained from a public repository. The dendrogram of each reveals a complex population structure, where *Acinetobacter* species isolates from Lagos, Nigeria, are distributed across multiple distinct clades rather than clustering as a single monophyletic group, suggesting multiple independent introductions of *Acinetobacter* species from the Nigerian ecosystem. For instance, within *A. junii* (Fig. 3), two discrete clusters emerged: one comprising isolates 24AR27665 (cattle rectal swab) and 24AR27668 (cattle nasal swab), which differ by 15 SNPs; and another formed by isolates 24AR27669 (cattle nasal swab) and 24AR27791 (human urine), differing by 17 SNPs. Isolate 24AR27701 remained unclustered, its nearest neighbor separated by 23 SNPs. All five *A. junii* isolates exhibit extensive divergence, approximately 48,355 SNPs from the reference genome GCF_018336855.1 and 24,000–32,000 SNPs relative to 43 global *A. junii* strains (Fig. 3). However, public repository sequence SRR21472687 recovered from remnant water in Pakistan was observed to be the closest to the five *A. junii* strains with SNP differences between 24,822 and 24,827. Similarly, analysis of fourteen *A. nosocomialis* isolates identified two principal clusters of tightly related strains (Fig. 4). Cluster A centers on human blood isolates 24AR27772 and 24AR27771 alongside human oral swab isolate 24AR27773; these share very close ties to cattle nasal swab isolate 24AR27662 (8 SNPs) and to cattle nasal swab 24AR27661, cattle rectal swab 24AR27700, and human blood 24AR27770 (13–22 SNPs), consistent with recent transmission events. Cluster B includes abattoir effluent isolate 24AR27704, human sputum isolate 24AR27774, and human blood isolate 24AR27892, separated by 10–20 SNPs, with an additional 17-SNP link to 24AR27700. Isolate 24AR27683 (human blood) stands apart as a distant outlier (117–155 SNPs). All *A. nosocomialis* isolates diverge by roughly 69,000 SNPs from the reference genome GCF_041021905 and 40,000–60,000 SNPs from the other global strains, except public strain ERR10441276 isolated from human urine in Ouagadougou, Burkina Faso was observed to be the closest to the sequenced *A. nosocomialis* with SNP differences between 593 and 604, indicating overall species divergence alongside variable epidemiological linkages (Fig. 4). However, pairwise comparisons further underscored specific affinities: *A. nosocomialis* isolates 24AR27662 (cattle nasal swab) and 24AR27772 (human bloods) differ by 8 SNPs, while isolates 24AR27704 (abattoir effluent) and 24AR27774 (human sputum) differ by 10 SNPs. Among *A. junii*, isolates 24AR27669 (cattle nasal swab) and 24AR27791 (human urine) are separated by 17 SNPs. Notably, interspecies analysis revealed a cluster of five isolates—three *A. nosocomialis* (24AR27662, 24AR27772, 24AR27704) and two *A. junii* (24AR27665, 24AR27668)—that differ by only 15 SNPs, suggestive of shared evolutionary or transmission dynamics warranting deeper investigation.

## 4 Discussion

In this study, WGS of 19 *Acinetobacter* species isolates from human, animal, and environmental sources in Lagos resolved taxonomic ambiguities, enabling precise strain differentiation and the identification of AMR markers, which is vital given the genus *Acinetobacter* genomic plasticity. Notably, 22 non-*Acinetobacter* isolates were detected in this study using MALDI-TOF, primarily *Achromobacter* spp. and *Stenotrophomonas maltophilia*, which are emerging pathogens associated with nosocomial infections [46], [47]. Their detection in clinical and abattoir samples indicates shared reservoirs, as seen in prior reports from slaughterhouse effluents and contaminated medical devices [46], [47]. The presence of *Achromobacter* and *Ochrobactrum* in food animals underscores the need for broader One Health surveillance, as these genera are underrepresented in Nigeria's AMR frameworks.

The AMR profiles of *Acinetobacter* isolates from Lagos exhibit similarities and differences with global trends. The 100% resistance of *Acinetobacter* species isolates to cefotaxime recorded in this study likely reflects selective pressures from the use of antibiotics in healthcare and agriculture, which promotes horizontal gene transfer, as observed in Iranian strains [48]. Similarly, the complete resistance to fosfomycin observed in this study aligns with fosfomycin-resistant *Acinetobacter* species documented by Sharma et al. [49]. This study further documented 100% (14/14) of *A. nosocomialis* resistance to chloramphenicol which contrasts with full susceptibility in *A. junii*, highlighting species-specific mechanisms amid genetic heterogeneity [50], [51]. All *A. nosocomialis* strains are multidrug-resistant strains, as they are resistant to at least three antibiotic classes [52]. However, the complete aminoglycoside susceptibility (e.g., amikacin) of the *Acinetobacter* species recorded in this study aligns with higher reported efficacy (50–86.7%) previous described by Castanheira et al. [50]. On the other hand, the intermediate resistance to ciprofloxacin by the 19 *Acinetobacter* isolates reflect the dose-dependent effects of fluoroquinolones from widespread use [48]. However, the singular instance of colistin resistance, notably from *A. junii* (24AR27669), recovered from ready-to-slaughter cattle, diverges from findings in regions such as parts of Asia, where colistin resistance has been increasingly alarming due to different selective pressures associated with hospital versus agricultural use [53]. Interestingly, all *Acinetobacter* isolates are susceptible to 12 of 17 tested antibiotics, including imipenem, meropenem, and tigecycline in this study, which contrasts with a greater than 90% resistance rate documented in regions such as the Middle East, Southern Europe, and North Africa, likely due to variations in antibiotic usage practices, stewardship policies, and healthcare infrastructure [50], [53], [54], [55].

Furthermore, the genomic analysis of *Acinetobacter* species identified four cephalosporin resistance genes in *A. nosocomialis*, including variants *bla*_ADC-148_, *bla*_ADC_, *bla*_ADC-25_, and *bla*_ADC-68_—class C β-lactamases targeting broad-spectrum cephalosporins and monobactams [56], [57]. Multiple ADC alleles per strain imply horizontal gene transfer or recombination [58]. Globally, *bla*_ADC-68_ and *bla*_ADC-148_ have been reported in *Acinetobacter* species isolates from Italy [59] and China [60]. The presence of both historic (*bla*_ADC-25_) and emerging (*bla*_ADC-148_) alleles in *A. nosocomialis* isolated from Lagos suggests a convergence of local and imported resistance determinants, potentially facilitated by medical tourism and travel among healthcare workers. In sub-Saharan Africa, *bla*_ADC-25_ has been identified in *A. haemolyticus* from Uganda, in conjunction with *bla*_OXA-264_ and *sul2*, via mobile genetic elements such as *Tn125* [61]. Although predominantly chromosomal, *bla*_ADC_ genes can integrate into plasmids via ISAba1-flanked transposons, enabling interspecies spread in high-density settings [62]. The detection of these genes in Lagos *A. nosocomialis* isolates illustrates *Acinetobacters'* adaptability to local and global selective pressures, necessitating enhanced stewardship, WGS surveillance, and international collaboration to limit multidrug-resistant clone dissemination. Additionally, 17 efflux pump genes were observed in *A. nosocomialis* from this study, which are known to encode for resistance against β-lactams, aminoglycosides, fluoroquinolones, tetracyclines, and carbapenems, while also enhancing biofilm formation and motility. Specifically, the *Ade*IJK efflux pump system, which encodes resistance against β-lactams, chloramphenicol, tigecycline, and trimethoprim, contributing to persistence documented in this study, was recorded in Italian *A. nosocomialis* strains [63]. Additionally, AdeFGH efflux pump system which targets aminoglycosides, fluoroquinolones, tetracyclines, and macrolides, and enhancing virulence recorded in this study, has been documented in *A. nosocomialis* in China [64]. Furthermore, the *abe*S gene, belonging to the SMR family recorded in this study, is known to expel antibiotics, which is consistent with reports from the Middle East [59], [65]. The regulatory efflux pump genes *ade*S and *ade*R, along with *Ade*ABC components, contribute to multidrug resistance observed in this study, mirrors trends reported in the United States [63] and Mexico [66]. The MFS pumps (*ade*M, *amv*A, *aba*Q), which further promote resilience and biofilm formation, were detected in all *A. nosocomialis* isolates in this study, correlating with pathogenicity in clinical settings documented in the USA [63] and Europe [62]. These mechanisms exacerbate treatment challenges and cross-reservoir transmission risks in urban environments like Lagos.

The virulence gene profiles revealed pronounced differences between *A. nosocomialis* and *A. junii*, indicating distinct pathogenic adaptations. *A. nosocomialis* harbored 68 genes across seven categories, emphasizing its opportunistic nature through exotoxins and biofilms that enable colonization and immune evasion [67]. In contrast, *A. junii* possessed only six genes across five categories, lacking exotoxin elements and biofilm, consistent with its lower environmental risk [68]. Conserved genes like *omp*A (immune modulation and biofilm), *ACIU_RS00500* (nutrient metabolism), *bfm*R (regulation), *pil*T and *pil*G (adherence), and *vgrG/tssl* (effector delivery) across both *Acinetobacter* species documented in this study, suggest foundational roles in pathogenesis, offering potential diagnostic and therapeutic targets despite challenges in specificity [69], [70]. Additionally, plasmids R3-T21 and R3-T60 in *A. nosocomialis*, absent in *A. junii*, observed in this study, highlight the genus-specific dynamics of horizontal gene transfer, even in the absence of overt AMR or virulence determinants [71]. These Rep3-family replicons form discrete lineages with greater than 95% identity at the rep loci, potentially serving as niche-adapted vectors for resistance [72]. This finding underscores the need for vigilant genomic surveillance, since even genus-specific plasmid replicons lacking overt resistance or virulence genes can act as niche-adapted vectors that facilitate the emergence and spread of antimicrobial resistance [72].

The SNP-based phylogenetic analysis of 19 Nigerian *Acinetobacter* isolates reveals a polyphyletic distribution among 106 global strains, indicative of multiple independent introduction and underscoring heterogeneous phylogenies that emphasize ecological and cross-host linkages. For *A. nosocomialis*, the formation of two clusters with low intra-cluster SNP variances (8–22 SNPs) and one outlier (117–155 SNPs), alongside divergences of approximately 69,000 SNPs from the reference genome GCF_041021905 and 40,000–60,000 SNPs from global strains, suggest distinct evolutionary lineage and a reflection of species-level divergence and regional genomic uniqueness [65]. This finding aligns with Nemec et al. [73], who documented phenotypic and genotypic diversity in global isolates with evidence of divergence, though their emphasis on taxonomic delineation contrasts with the multi-source transmission dynamics observed in this study. In contrast, *A. junii* exhibits two clusters (15–17 SNPs within) and one unclustered isolate (23 SNPs from the nearest), with high divergence (∼48,355 SNPs from the reference genome GCF_018336855.1) and 25,000–32,000 SNPs from global strains, indicating substantial genomic divergence [21]. This result contrasts with the findings of Krizova et al. [74], whose Czech clinical and environmental isolates displayed moderate SNP variability (10,000–20,000 from references) within contained lineages, thereby implying that the Nigerian strains reflect a more dynamic, ecosystem-driven evolution influenced by animal-human interfaces not as prominently featured in European datasets. Additionally, the interspecies clustering of five isolates (three *A. nosocomialis* and two *A. junii*) differing by only 15 SNPs suggests possible horizontal transfer and shared ecological niche [21], [65]. Like other ESKAPE pathogens in African countries [[75], [76]]. These findings illuminate zoonotic and environmental interconnections in *A. nosocomialis* and *A. junii*, posing significant public health risks in resource-constrained settings like Nigeria, where non-human reservoirs such as abattoirs may precipitate opportunistic human infections and outbreaks; consequently, enhanced surveillance of these reservoirs, bolstered abattoir hygiene protocols, and integrated genomic tracking are imperative to disrupt transmission pathways, in alignment with One Health frameworks aimed at mitigating infection burdens in vulnerable populations as Lagos, State Nigeria.

## 5 Conclusion

This study provides a comprehensive characterization of *Acinetobacter* species isolated from human, animal, and environmental sources in Lagos, Nigeria, by leveraging a multifaceted approach that combines phenotypic identification, MALDI-TOF mass spectrometry, and WGS. Of the 2835 samples collected between April 2023 and March 2024, 19 isolates were confirmed to be *Acinetobacter*, comprising 14 *A. nosocomialis* and five *A. junii*. Antibiotic susceptibility testing revealed 100% resistance to cefotaxime and fosfomycin across both species, with 100% of *A. nosocomialis* additionally resistant to chloramphenicol, underscoring the persistent MDR threat, posed by these pathogens in a One Health context. Genomic analyses elucidated species-specific resistance and virulence profiles, with *A. nosocomialis* harboring 21 intrinsic AMR genes, including four distinct *bla*_ADC_ variants and 17 efflux-related genes, that were absent in *A. junii*. Virulence determinants were markedly more extensive in *A. nosocomialis*, encompassing 68 genes across seven mechanisms, compared with only six in *A. junii*, which lacked exotoxin and biofilm formation pathways. Specifically, both species shared six conserved virulence-associated genes (*ompA, ACICU_RS00500, bfmR, pilG, pilT,* and *vgrG/tssI*), highlighting potential core mechanisms underlying pathogenicity. The exclusive presence of two distinct plasmids in *A. nosocomialis* further suggests enhanced adaptive capabilities in this species. Phylogenomic evaluation demonstrated the formation of distinct sub-clusters among isolates from diverse sources, with evidence of close genetic relatedness (e.g., clusters differing by as few as 15 single nucleotide polymorphisms [SNPs] across human, animal, and environmental origins), indicative of potential inter-source transmission and cross-species dynamics. These findings emphasize the evolutionary interconnectedness of *Acinetobacter* populations within Nigeria's One Health sector and the risk of MDR strain dissemination. The results of this study advocate integrating advanced molecular diagnostics, such as WGS, into routine surveillance to monitor AMR and virulence evolution. Future efforts should prioritize longitudinal studies to delineate transmission pathways and inform targeted interventions, thereby mitigating the public health burden of these opportunistic pathogens in resource-limited settings like Nigeria.

## CRediT authorship contribution statement

**Samuel O. Ajoseh:** Writing – original draft, Visualization, Methodology, Investigation, Formal analysis. **Abdul-Azeez A. Anjorin:** Writing – review & editing. **Hanka Brangsch:** Writing – review & editing, Validation, Software, Formal analysis, Data curation. **Heinrich Neubauer:** Writing – review & editing, Validation, Resources, Project administration, Funding acquisition. **Gamal Wareth:** Writing – review & editing, Validation, Supervision, Resources, Project administration, Methodology, Investigation, Funding acquisition. **Kabiru O. Akinyemi:** Writing – review & editing, Validation, Supervision, Project administration, Methodology, Investigation, Funding acquisition, Conceptualization.

## Source of funding

Open access and manuscript preparation funding were facilitated through Projekt DEAL. The Friedrich-Loeffler-Institut (Federal Research Institute for Animal Health, FLI), 07743 Jena, Germany, provided financial support for open access publication and manuscript preparation.

## Declaration of competing interest

To The Editor-in-Chief.

The authors declare that this manuscript has not been published elsewhere nor currently being considered in another journal. The authors have no known competing financial interests or personal relationships that could have appeared to influence the work reported in this paper. All funding and institutional support relevant to this study has been transparently acknowledged in the manuscript.

## Data Availability

Raw sequencing data have been uploaded to the European Nucleotide Archive (ENA) and are publicly available under the BioProject PRJEB97386.

## Appendix A Supplementary data

Assembly data of Acinetobacter junii and Acinetobacter nosocomialis isolated from humans, animals, and the environment in Lagos, Nigeria. Image 1
